# The expected economic burden on the healthcare system because of quarantining patients with monkeypox virus

**DOI:** 10.15537/smj.2023.44.3.20220515

**Published:** 2023-03

**Authors:** Ali Alshahrani

**Affiliations:** *From the Department of Clinical Pharmacy, College of Pharmacy, Tiaf University, Taif, Kingdom of Saudi Arabia.*

**Keywords:** economic burden, monkeypox, quarantine, healthcare system

## Abstract

Quarantine is a common public health intervention that is often used to curb pandemics of infectious diseases. Quarantine is the intentional separation of people who are either suspected or confirmed to be infected with a contagious virus from the uninfected population. The goal of this study was to determine the expected economic cost for healthcare systems due to quarantine in the case of the monkeypox virus. A systematic literature review of studies on similar virus outbreaks was performed. The findings affirm that quarantine effectively mitigates the spread of a virus outbreak, but it has high direct and indirect costs that can only be justifiable for a dangerous virus with high mortality. The monkeypox virus presents moderate risk, unlike high-risk diseases for which quarantine is mandatory. The study recommends the introduction of mass vaccination programs and public awareness and sensitization forums to inform the population about the best behavioral practices to curb the spread of monkeypox virus.

**S**ince May 13, 2022, monkeypox infections have been reported in various nations worldwide, affecting more people outside of Africa than ever before. As of June 6, 2022, a total of 1019 cases of monkeypox were reported from 29 nations.^1^ Regions such as North America, Europe, and Australia, where the virus is not endemic, have reported alarming cases. To date, the United Kingdom (UK) has reported a majority of the infections, with 302 suspected and confirmed cases, followed by Spain (198 cases), Portugal (153 cases), Canada (80 cases), and the United States (US) (30 cases).^1^

Monkeypox is a viral zoonosis that occurs when a person or animal is exposed to the monkeypox virus. Symptoms include rashes, swelling, head and muscle aches, fever, and back pain.^2^ Monkeypox is prevalent in its endemic regions of West and Central Africa. Countries such as the Democratic Republic of Congo (which has the highest infection rate), Central African Republic, Cameroon, Ivory Coast, Liberia, Gabon, the Democratic Republic of the Congo, Nigeria, and Sierra Leone have reported high rates of infections in the past. Although epidemiological investigations are still in progress, researchers have not correlated current infections with travel links to endemic areas. Consequently, the uncharacteristic spread of monkeypox outside of Africa with no travel links to the continent has created a sense of panic that the virus is now spreading globally.

The Centers for Disease Control and Prevention (CDC) in the US reported that the risk to the general public is currently low, but they encourage people to avoid close contact with those infected. These include people with genital and skin lesions, as well as infected or dead animals. The CDC also urges people exhibiting symptoms of the virus, such as unexplained skin rashes or lesions, to isolate themselves and reach out to the closest healthcare provider for guidance.

Current available evidence from the World Health Organization (WHO) suggests the risk is greatest for those who have made close physical contact with those infected with the virus while they are symptomatic.^3^ The WHO recommends that people with suspected or confirmed infection be promptly isolated in a room with sufficient ventilation, a clean and dedicated bathroom, and staff. The CDC advises that isolation precautions be continued until all lesions have resolved, the scabs have eroded, and a new layer of skin has grown.^4^

On May 20, 2022, Belgium became the first nation to enact a compulsory 21-day quarantine for patients confirmed to have been infected by the monkeypox virus.^1^ The UK and Germany followed suit soon afterward, adding that people with a high risk of contracting the virus should self-isolate for at least 21 days. The UK Health Security Agency (UKHSA) notes that individuals like sexual partners, persons living within the same house, or any other person coming in contact with an infected person’s bodily fluids (notably sneeze or cough) should self-isolate.^1^

However, critics have described the move to quarantine monkeypox patients as unnecessary. President Joe Biden recently said that quarantines are not necessary to mitigate the spread of the monkeypox virus in the US.5 He further explained that the new pandemic does not warrant the same kind of concern as in the case of COVID-19.^5^ The president and government officials downplayed the spread and expected the impact of monkeypox to be minimal, especially provided that it has been monitored for decades and has existing vaccine doses and medications. The purpose of the current study was to investigate the expected economic cost for healthcare systems due to quarantine in the case of the monkeypox virus through a systematic review of literature.

## Methods

The current study was a systematic review of current literature. The goal was to find research the estimated economic cost burden for healthcare systems due to quarantine in the case of monkeypox virus outbreak. Different previous studies that focused on different virus outbreaks where quarantining was used as the primary virus mitigation method were reviewed. Conclusions were arrived at on the basis of the information found from the various studies and resources that were reviewed.

### Data collection

The articles used in this literature review were obtained from different academic databases and peer reviewed open-source online research publishing websites. A total of 38 resources were first collected and after evaluation for their suitability in creating the necessary knowledge and findings for this study, only 29 of these were suitable for use in forming the basis for the findings of the current study.

### Literature review and findings

Different authors have researched the cost of quarantine, particularly the economic impact on the different aspects of the healthcare system. Despite these studies, there has not been a study that compares the overall economic impact of the monkeypox quarantine as the current study sought to investigate. Below is a systematic analysis of literature focused on the cost and benefit of quarantine strategies and the impact on the healthcare sector.

### Effect of quarantine on infection rates

In a pandemic, quarantine and similar social distancing strategies are often enforced to curb viral spread, particularly when there are no vaccines or medicines to manage symptoms.^6^ In general, quarantine strategies have effectively reduced infection rates and delayed new infections over time.^7^ After modeling the 2003 severe acute respiratory syndrome (SARS) pandemic, Hsieh et al^8^ determined that quarantine prevented about 461 additional SARS infections and 62 more deaths in Taiwan. After studying SARS data from Hong Kong, Riley et al^9^ determined that public health interventions implemented during the SARS outbreak effectively reduced the contact rates among infectious people and the entire population.

Marshall evaluated the impact of quarantine on the infection and modified the SIR, a conventional epidemiology model used to mathematically simulate the spread of infectious diseases. The findings revealed that quarantines drastically minimize the rates of infection, thus limiting spikes that can overwhelm the health system.^10^ Importantly, the research also showed that a reduced infection rate has a strong positive correlation with socioeconomic status.^10^ Indeed, quarantine is an effective measure to mitigate the spread of an infectious virus/disease. While quarantines have proved effective in curbing viral infections, critics have questioned the economic feasibility of such measures. Hence, it is vital to perform a cost-benefit analysis to assess the efficacy of quarantine in responding to the current monkeypox outbreak.

### Direct financial impact of quarantine

Direct healthcare costs include those associated with the creation and implementation of control programs, such as facility construction and frontline workers’ salaries.^11^ In research to determine the economic burden of COVID-19 in China, Jin et al^12^ calculated the unit costs of treatment in different regions using the healthcare industry salary index. The State Council^12^ reported that a total of 42,600 frontline workers attended to suspected or confirmed COVID-19 cases.The researchers estimated that the average risk subsidy per day for every frontline professional was Yen (¥) 300.Moreover, the researchers estimated that the healthcare cost of suspected cases of COVID-19 was ¥584.08 (US dollars [$] 84.53) for every individual. The average cost for the treatment of an individual confirmed to have contracted the virus was ¥22,061.94. The researchers calculated the routine healthcare expenses to be $0.31 billion. Inpatient treatment constituted 44.2% of routine healthcare expenses, while medications represented 32.5% (¥0.70 billion/¥2.15 billion). Overall, the healthcare expenses of the outbreak of COVID-19 in China for the initial 3 months of 2020 were estimated to amount to 0.62 billion USD.^12^

Gamage et al^13^ assessed the direct and indirect costs incurred due to quarantine for COVID-19 in Sri Lanka. The direct cost of quarantine was determined to be Rupees (Rs) 1610 per patient every day, and accommodation expenses accounted for 54% of the costs. The mean income loss from quarantining an informal sector worker for 14 days was Rs 13,800, and for a government-sector worker was Rs 30, 000. The loss of output as a result of placing primary contacts in quarantine was Rs 166 billion. The researchers concluded that as a disease evolves, quarantine guidelines must accommodate resource availability and disease burden.

Ghaffari et al^14^ sought to calculate both the indirect and direct expenses of treating COVID-19 patients within a referral hospital setting and the economic burden of the COVID-19 pandemic in Iran in 2020.They concluded that the direct healthcare expenses amounted to $1,791,172, averaging $3,755 per person.^14^ Mubayi et al^15^ studied the relationship between quarantine, isolation, and financial implications using dynamical models. They compared 3 distinct quarantine methods implemented in addition to a single isolation approach, and analysis proved that a single policy (either quarantine or isolation) was enough to control an outbreak. Since isolation costs more than quarantine, mainly due to the cost and time spent constructing isolating facilities, the researchers recommended using a joint quarantine-isolation policy.

Those supporting quarantine measures suggest that such measures have a positive economic impact since expenses incurred lessen direct and indirect costs in the future. Gupta, Moyer, and Stern assessed the economic implications that Toronto experienced following the implementation of quarantine for patients suspected or confirmed to have SARS in the 2003 outbreak.^16^ In order do so, they compared the associated costs of quarantine in 2 different outbreak cases. In the first scenario, SARS was allowed to propagate throughout the study population without taking mitigation intervention. In the second scenario, quarantine was implemented at the beginning of the outbreak to contain the virus.^16^ The latter proved more rational, and the authors concluded that quarantine is effective in slowing the spread of infectious outbreaks while reducing expenditure compared to taking a passive approach.^16^

Ontario’s finances for the first quarter of the 2003-2004 fiscal year indicate that the provincial government incurred costs amounting to $10 million for administrative activities for the containment of the SARS virus.^16^ The government spent an additional $1 million for protecting the jobs of individuals who had been quarantined. The government also had to set up an assistance office for SARS to look into the interests of quarantined or isolated employees, which came at a cost of $1 million.^16^ From these figures, Gupta at al^16^ estimated the direct expenses of the epidemic to be $12 million. On average, the study findings indicated that a SARS patient spent approximately 14 days in the hospital.^17^ The average cost of a single night in the intensive care unit in Canada was estimated to be $1,836.16 Findings from the study revealed that contagious and infectious diseases can effectively be contained through quarantine, which also reduces costs compared to not adopting any containment measure.^16^

### Indirect financial impact of quarantine

Indirect cost to the healthcare system comprises productivity losses from quarantining patients as well as restriction of movement of people who have not contracted the disease. Jin et al^12^ estimated the productivity losses from COVID-19 in China to amount to ¥2641.61 billion (US $ 382.29 billion). This loss was attributed to the lost working time as the population’s movement was restricted, regardless of their status. The productivity loss was highest in areas with the greatest numbers of employed people, such as Guangdong, Jiangsu, and Beijing.^12^ The researchers concluded that contact tracing and quarantine measures reduce the number of infections but result in great inconvenience to the general population, as well as subsequent indirect costs.^11^

**Table 1 -:** Summary/Illustration of the direct costs.

Researcher	Disease	Daily cost per person ($)
Jin et al^12^	COVID-19	$84.53
Gamage et al^13^	COVID-19	$20
Ghaffari et al^14^	COVID-19	$268
Mubayi et al^15^	SARS	$131
COVID-19: corona virus disease-19, SARS: severe acute respiratory syndrome		

Ghaffari et al^14^ calculated average indirect expenses, particularly those associated with income that is lost due to premature death, the productivity lost due to a patient being hospitalized, and absenteeism from work due by COVID-19. They estimated the cost to be $11,634 per person. The inpatient costs at the national level amounted to $1,439,083,784.^14^

Gupta et al^16^ measured the costs indirectly associated with SARS by reviewing the productivity that is lost within the economy or the opportunity cost of the disease. In Ontario, the income per capita was valued at $30,702. These costs were quantified as lost productivity after people who were exposed failed to attend work for at least 10 days. In Toronto, the researchers successfully used the average daily wage to value the loss of productivity at $1140 for every individual isolated person. Using a mortality rate of 11% and the average life expectancy of 71 years in Canada, the researchers computed society’s economic cost per premature death.^16^

They estimated that the average age of death was 56 years. Consequently, this indicated a mean lost life time of 15 years. They examined average worker wages in Ontario and came up with an average of $30,702 lost for every worker per every year of life. Hence, the mortality of SARS led to productivity loss with approximately $460,530 for every single life that was lost. Hence, the researchers concluded that quarantine would help to minimize the cost that the economy would incur from premature deaths.^16^

## Discussion

### Role of the national government and healthcare sector during a pandemic

During a healthcare pandemic, the government has a responsibility to protect its citizens from the various adverse effects of the disease outbreak. During public health crises and the cascading social and economic consequences, governments are required to respond swiftly and effectively to a series of challenges.^18^ Common issues to address include coordinating emergency responses and establishing necessary quarantine measures. The health sector (including public and private healthcare facilities) plays a role in advocacy and leadership in the preparedness and response efforts geared towards managing the pandemic.^19^ In coordination with other departments and in support of the central government, the health department creates awareness about the risks and the likely health consequences of the outbreak and provides leadership and guidance on the required measures. As Berman explains, efforts at the federal level include.^20^

- Stockpiling supplies such as vaccines, drugs, and medical equipment
- Testing and diagnostic development of innovative equipment and medications
- Funding research for vaccines and antivirals
- Facilitating public education on best practices with regard to the pandemic

Direct versus (vs.) indirect cost of quarantine on the healthcare system. Generally, economic researchers quantify the cost of a pandemic with the inclusion of direct expenditures, the indirect health and non-health costs, and the fall in GDP. Direct healthcare costs include protective equipment, vaccines, medication, and contact tracing, and testing. Direct costs also include the construction and maintenance of quarantine facilities, as well as special measures enforced by institutions to prevent the spread of infections.

Indirect costs include productivity losses from mortality, morbidity, and quarantines. In the US, the indirect cost of COVID-19 was estimated to be approximately $13 trillion (90% of the annual national GDP).^21^ Mulligan also estimated an annual welfare loss of $7 billion in the US.^22^ Another study determined that in the US, an estimated cost amounting to $286,000 was incurred.^23^

### Cost-benefit analysis of interventions

Analysts have sought to compare the cost of intervention strategies implemented to curb the spread of infections against the benefits yielded from such strategies. López-Valcárcel and Vallejo-Torres^24^ sought to estimate the cost of the COVID-19 pandemic in Spain. They considered the macroeconomic losses of foregone GDP and the direct and indirect expenses of prevention and treatment. They explain that Spain was among the most-affected nations with a macroeconomic cost linked to COVID-19 amounting to 24% of the GDP in 2019.^24^ Nonetheless, they determined that the direct health costs of the pandemic were only an insignificant portion of the total cost of the pandemic.^24^

### Monkeypox virus vs. other outbreaks

Each virus has unique spreading patterns and mortality rates. Hence, interventions should be adopted rationally to effectively curb the spread and deaths caused by the virus while also economically protecting the healthcare sector and society. Compared to other virus pandemics, the current monkeypox outbreak only poses a reasonable risk to general public health across the world. Monkeypox has estimated transmission rates of 3.3% to 30%, while its fatality rate is estimated to be between 1% and 10%.^25^ Monkeypox triggers a milder illness as compared to smallpox, which is fatal in approximately 30% of cases.^26^ The WHO estimates the general rate of fatalities for SARS patients at 14% to 15%.^27^ The COVID-19 statistics differ slightly among nations, but the average mortality rate is 2.7%, and the infection rate stands at 2%.^28^

Conclusion, despite the transmission rate of the monkeypox virus being high compared to other virus infections, the current risk to the general public remains low. A majority of infected people report relatively mild symptoms that do not create an urgent need for hospitalization. Many reviewed studies affirm that quarantine strategies effectively reduce the infection rate and delay new infections over time, but the healthcare departments of affected nations have to incur huge expenses, particularly from the indirect costs of quarantine. While a significant budget is required to cater to the direct expenses of quarantine, the mentioned studies show that the indirect costs of quarantine by far exceed the direct costs.

Those supporting quarantine measures suggest that such measures have a positive economic impact since expenses incurred in the present reduce future direct and indirect costs.^16^ However; this depends on the rate of spread of the disease and its mortality rate. Non-pharmaceutical interventions such as quarantines are the main strategies used for ensuring that the spread of the disease is under control when there are no vaccines or clinical treatment for specific illnesses.^28^ However, since there are known and effective vaccines and medications to prevent and cure monkeypox, it is unjustifiable to incur exorbitant costs in quarantining infected people or their close contacts. The cost of quarantine of close contacts includes both direct and indirect costs.

As such, although quarantine would effectively mitigate the spread of the current strain of the monkeypox virus, it would cause significant economic disruptions to the world economy and healthcare ministries. Indeed, quarantining suspected people would adversely affect the GDPs of affected nations and cause immense productivity losses. Instead, governments across the world (including non-endemic counties) should immediately initiate mass vaccination programs to protect their populations. The WHO explains that the smallpox vaccine is 85% effective in preventing monkeypox infections. Rather than calling for isolation and quarantine programs, other nations should emulate the US, which has already ordered 13 million doses of the vaccines.^29^ In addition, public awareness and sensitization forums should be held to inform the population about the best behavioral practices, such as handling potentially infectious animals and following routine hygiene practices.
